# Age- and Sex-Differences in Cardiac Characteristics Determined by Echocardiography in Masters Athletes

**DOI:** 10.3389/fphys.2020.630148

**Published:** 2021-01-18

**Authors:** Savannah V. Wooten, Stefan Moestl, Phil Chilibeck, José Ramón Alvero Cruz, Uwe Mittag, Jens Tank, Hirofumi Tanaka, Jörn Rittweger, Fabian Hoffmann

**Affiliations:** ^1^Department of Kinesiology and Health Education, The University of Texas at Austin, Austin, TX, United States; ^2^Institute of Aerospace Medicine, German Aerospace Center, University of Cologne, Cologne, Germany; ^3^Physical Activity Complex, University of Saskatchewan College of Kinesiology, Saskatoon, SK, Canada; ^4^Human Physiology and Physical Education and Sports, University of Malaga, Málaga, Spain; ^5^Department of Cardiology, University Hospital Cologne, Cologne, Germany

**Keywords:** aging athlete, cardiac function, cardiac morphology, healthy ageing, echocardiogaphy, sex difference

## Abstract

**Background:**

Cardiac function and morphology are known to differ between men and women. Sex differences seen with echocardiography have not been studied systematically in masters athletes.

**Purpose:**

To evaluate sex differences in cardiac structure, function and left ventricular (LV) systolic global longitudinal strain among masters athletes.

**Methods:**

This cross-sectional study comprises of 163 masters athletes (*M* = 109, 60 ± 12 years; *F* = 55, 57 ± 12 years, range 36–91 years) who participated at the 23rd World Masters Athletics Championship held in Málaga, Spain. All athletes underwent state-of-the-art echocardiography including cardiac function, morphology, strain and hemodynamic assessment.

**Results:**

Left ventricular mass was higher in male than in female athletes (174 ± 44 vs. 141 ± 36 *g*, *p* < 0.01) due to greater end-diastolic intraventricular septal, LV posterior wall and LV basal diameter. However, LV mass index did not differ between the groups. End-diastolic LV volume and right ventricular area, both indexed to body-surface-area, were greater in men than in women (52.8 ± 11.0 vs. 46.1 ± 8.5 ml/m^2^, *p* < 0.01, 9.5 ± 2.4 vs. 8.1 ± 1.7 cm^2^/m^2^, *p* < 0.01). In contrast, women had higher LV systolic global longitudinal strain (-20.2 ± 2.6 vs. -18.8 ± 2.6%, *p* < 0.01) and LV outflow tract flow velocity (75.1 ± 11.1 vs. 71.2 ± 11.1 cm/s, *p* = 0.04). Systolic and diastolic blood pressure, LV ejection fraction, and stroke volume index were not different between sexes.

**Conclusion:**

Cardiac sex differences are present even among masters athletes. Lifelong exercise training does not appear to exasperate morphological difference to a point of cardiac risk or dysfunction in both male and female athletes.

## Introduction

Aging is associated with marked changes in heart function and morphology, also known as cardiac remodeling (Cheng et al., 2010). Left ventricular (LV) hypertrophy, aortic stiffening, and impaired diastolic function are age-associated changes known to be major contributors to age-related increases in cardiovascular disease risks (Cheng et al., 2009; Redheuil et al., 2011). In contrast, increased levels of physical activity are associated with lower cardiovascular morbidity and mortality (Maessen et al., 2016) and have been promoted to help counteract cardiovascular disease risks (WHO, 2018). Masters athletes are veteran and senior athletes ranging in age from 35 to above 100 years who regularly train at high levels for athletic competitions (Tanaka, 2012). Masters athletes are a model of primary or successful aging through the effects of lifelong exercise training (Tanaka et al., 2011). Chronic exercise training elicits a number of physiological adaptations that alter cardiac structure and function (Pelliccia et al., 1991) that are not only beneficial for sport performance but also valuable for overall cardiovascular health (Thompson et al., 2003; Maessen et al., 2016).

Emerging evidence from epidemiological and observational studies primarily in male athletes suggest that individuals who participated in lifelong strenuous exercise demonstrate an increased prevalence of atrial fibrillation, myocardial fibrosis, and coronary artery calcification (Möhlenkamp et al., 2008; Mozaffarian et al., 2008; Tanaka et al., 2011). Adverse changes in cardiac dimensions such as left ventricle morphology are among the most commonly observed among endurance trained individuals (Eijsvogels et al., 2016) although whether increased cardiac dimensions due to lifelong exercise training increase susceptibility to cardiovascular disease for certain individuals remains to be determined (Eijsvogels et al., 2016).

Cardiac function and morphology as well as cardiovascular risks are known to differ between men and women (Finocchiaro and Sharma, 2016). Even after normalization for body size, women demonstrate lower LV mass and higher ejection fraction than men (Petitto et al., 2018). Women also possess smaller blood volumes that contribute to smaller changes in systemic hemodynamics (Harms et al., 2011). Additionally, women develop greater diastolic dysfunction with aging, presumably, due to different hormones milieu on LV functional and morphological changes (Petitto et al., 2018). Female athletes also exhibit smaller absolute changes in cardiac dimensions when compared with male athletes (Finocchiaro and Sharma, 2016).

Current literature dealing with sex differences in cardiac structure and function has focused primarily on young and middle-aged competitive athletes (Pluim et al., 2000). Currently, there is no data that addresses sex differences in the effect of lifelong exercise on LV function and structure measured with echocardiography in masters athletes. Accordingly, the purpose of the present study was to identify sex differences in cardiac structure, function, and LV systolic global longitudinal peak strain in a group of lifelong exercising adults in track and field events.

## Materials and Methods

### Participants

Data collection took place at the 23rd World Masters Athletics Championships (WMAC) held in Málaga, Spain. A total of 163 masters athletes participated in the present study (108 male and 55 female athletes). Mean age was 58.8 years ranging from 36 to 91 years of age (Table 1). To be included in the study, individuals had to be registered and competing in the WMAC. This study was approved by ethic commission of Ärztekammer Nordrhein (lfd Nr 2018171). After informed consent was obtained, height and body weight of the participants were assessed with a stadiometer and balance scale, respectively, (MPE250K100HM, Kern und Sohn, Balingen, Germany).

**TABLE 1:** Sex differences in cardiac and hemodynamic measures between male and female masters athletes.

		Male	Female	Effect-size	*P*
Number	[n]	103	55	(Std. error)	
**General characteristics**					
Age	[y]	59.8 ± 12.3	56.8 ± 12.1	3.0 (2.0)	0.14
Height	[cm]	174.6 ± 6.8	163.8 ± 7.1	10.8 (1.2)	<0.01
Body mass index	[kg/m^2^]	24.5 ± 3.1	23.3 ± 3.6	1.3 (0.6)	0.02
Body surface area	[m^2^]	1.90 ± 0.18	1.68 ± 0.15	0.22 (0.03)	<0.01
**Exercise modality**					
Endurance	[%]	39.8	41.8		
Sprint	[%]	38.0	30.9		
Strength	[%]	22.2	27.3		
**Age group composition**					
35–44 years	[n (%)]	12 (12%)	9 (16%)		
45–54 years	[n (%)]	24 (23%)	16 (29%)		
55–64 years	[n (%)]	31 (30%)	15 (27%)		
65–74 years	[n (%)]	21 (20%)	10 (18%)		
>74 years	[n (%)]	15 (15%)	5 (9%)		
**Hemodynamics**					
Heart rate at rest	[1/min]	59.4 ± 10.5	62.9 ± 11.2	−3.5 (1.8)	0.06
Systolic blood pressure	[mmHg]	129 ± 14	127 ± 17	2.1 (2.5)	0.40
Diastolic blood pressure	[mmHg]	79 ± 8	77 ± 8	2.0 (1.3)	0.14
Mean arterial pressure	[mmHg]	96 ± 9	93 ± 9	2.8 (1.5)	0.12
Cardiac output	[l/min]	5.7 ± 1.4	5.1 ± 1.3	0.5 (0.3)	0.05
Systemic vascular resistance	[Wood Unit]	17.8 ± 4.8	19.2 ± 4.4	−1.3 (0.9)	0.14
**Morphology**					
IVSd	[mm]	11.3 ± 2.2	10.2 ± 2.0	1.1 (0.3)	<0.01
LVend-diastolic diameter	[mm]	44.4 ± 4.8	41.9 ± 4.6	2.6 (0.8)	<0.01
LVPWd	[mm]	10.7 ± 1.9	10.1 ± 1.7	0.6 (0.3)	0.05
LV mass	[g]	174 ± 44	141 ± 36	32.6 (6.5)	<0.01
LVmass index	[g/m^2^]	91.6 ± 23.9	84.6 ± 23.0	7.1 (3.9)	0.07
LV end-systolic volume index	[ml/m^2^]	20.6 ± 4.6	17.3 ± 3.8	3.2 (0.8)	<0.01
LVend-diastolic volume index	[ml/m^2^]	52.8 ± 11.0	46.1 ± 8.5	6.8 (1.7)	<0.01
RVend-systolic area index	[cm^2^/m^2^]	5.2 ± 1.4	4.0 ± 1.0	1.1 (0.2)	<0.01
RVend-diastolic area index	[cm^2^/m^2^]	9.5 ± 2.4	8.1 ± 1.7	1.4 (0.3)	<0.01
**Systolic function**					
LV ejection fraction	[%]	60.7 ± 5.1	62.4 ± 4.7	−1.7 (0.9)	0.06
RV fractional area change	[%]	43.5 ± 18.5	49.5 ± 9.1	−5.9 (1.4)	0.07
TAPSE	[mm]	29.4 ± 4.4	29.1 ± 5.2	0.4 (0.8)	0.63
LV peak dispersion	[ms]	62.1 ± 28.4	50.2 ± 12.4	11.9 (4.1)	0.03
LV systolic global longitudinal peak strain	[%]	−18.8 ± 2.6	−20.2 ± 2.6	−1.4 (0.6)	0.01
LV OT mean velocity	[cm/s]	71.2 ± 11.1	75.1 ± 11.1	−3.9 (1.8)	0.04
LV OT stroke volume index	[ml/m^2^]	50.3 ± 10.7	48.7 ± 10.6	1.5 (2.0)	0.42
**LV Diastolic function**					
MV early filling wave (E)	[cm/s]	61.7 ± 19.7	74.4 ± 20.0	−12.7 (4.1)	<0.01
MVatrial filling wave (A)	[cm/s]	51.3 ± 20.2	58.5 ± 15.7	−7.1 (2.8)	0.02
E/A ratio		1.29 ± 0.40	1.35 ± 0.47	−0.6 (0.7)	0.38
MV averaged e prime	[cm/s]	10.3 ± 2.3	10.8 ± 2.8	−0.5 (0.4)	0.19
E/averaged e prime ratio		6.2 ± 2.2	7.2 ± 2.2	−0.9 (0.3)	0.01

*IVSd, intraventricular septal diameter; LV, left ventricular; PWd, posterior wall diameter; RV, right ventricular; TAPSE, tricuspid annulus plane systolic excursion; OT, outflow tract; MV, mitral valve; and e prime, Doppler velocities of the mitral annulus.*

On-site recruitment of the participants, study organization and data acquisition was supported by an adapted version of the REDCap electronic data capture tool (Harris et al., 2009, 2019). Subjects of this study were not involved in the design or conduct of this study. However, they played a crucial role in recruitment for our research.

### Echocardiography and Hemodynamic Assessment

Following a 10-min supine rest, participants underwent standard echocardiography procedures using a Vivid IQ ultrasound machine equipped with a MSC5 cardiac sector probe (General Electric, Boston, MA, United States) on an ultrasound stretcher with thoracic window. Clinical standard views were recorded including parasternal long and short axis (at the level of apex, papillary muscle, and aortic valve), 2-, 3-, 4-, and 5-chamber views. Morphological measurements included volumes and cardiac dimensions. Functional analysis was performed for systolic function (automated detection of ejection fraction), LV systolic global longitudinal peak strain by speckle tracking and stroke volume. For diastolic function, tissue Doppler spectra of the mitral annulus and pulsed wave Doppler spectra of transmitral filling were analyzed for early and atrial filling peak velocities as well as tissue velocities. Right ventricular function was measured by tricuspid annulus plane systolic excursion (TAPSE) and fractional area change (FAC). All echocardiography and hemodynamic examinations and analyses were performed by the same experienced cardiologist (FH, >700 readings).

End-systolic and end-diastolic LV volumes and right ventricular areas were indexed to body surface area. Left-ventricular mass was calculated by using the following equation: *LV Mass (g)* = *0.8 × 1.04 × [(LVEDD* + *IVSd* + *PWd)^3 – LVEDD^3]* + *0.6* (Devereux and Reichek, 1977; Kinno et al., 2016), where LVEDD = left ventricular end-diastolic diameter, IVSd = interventricular septal end-diastolic thickness, and PWd = posterior wall end-diastolic thickness. Stroke volume in the LV outflow tract was calculated using the following equation: *LVOT-SV (ml)* = *Π × (LVOT-Diameter/2)^2 × LVOT-VTI* (Flachskampf et al., 1990), where LVOT-VTI = velocity-time-integral of the pulsed-wave Doppler spectra in the LV outflow tract. Diastolic function of the left ventricle was assessed via multiparametric approach including transmitral filling patterns (early and atrial filling waves velocities), their ratios and the tissue Doppler velocities of the mitral annulus (e prime).

Heart rate and blood pressure were measured with an oscillometric upper arm cuff (CardioCube, Austrian Institute of Technology, Vienna, Austria). Measurements were repeated three times and averaged.

Moreover, systemic vascular resistance (SVR) and mean arterial pressure (MAP) were calculated using the following equations, respectively, *MAP (mmHg)* = (*systolic blood pressure* + *diastolic blood pressure × 2)/3* and *SVR (Wood Units)* = (*MAP – central venous pressure)/cardiac output*, whereas central venous pressure is derived from echocardiography.

### Statistical Analyses

Test of normality was used to check for normal distribution of data. Sex and age differences were compared using multivariate ANOVA (JMP 15.1.0, SAS Institute, Cary, NC, United States). Pearson’s correlation analyses were used to identify relationship between age and cardiac function and morphology within sex. In the case that the normality assumption was violated, Spearman rank correlation was used for correlation analyses. Data are expressed as means ± standard deviation (SD).

Power was calculated (Levine and Ensom, 2001; Rosner, 2015) retrospectively for exploratory endpoints IVSd, LV end diastolic volume indexed to body surface area, LV systolic global longitudinal peak strain and transmitral E-wave. Power values were found to be 88.9, 98.9, 88,9, and 96.9%, respectively, with given sample size.

## Results

### Characteristics and Hemodynamics

As shown in Table 1, age was not different between male and female athletes (*p* = 0.14). Male athletes had higher body mass index and body surface area than women (*p* = 0.02 and *p* < 0.01, respectively). Systolic and diastolic blood pressure were not significantly different between men and women (*p* = 0.40 and *p* = 0.14, respectively). Heart rate at rest was slightly higher in female athletes than in male counterparts (*p* = 0.06).

### Cardiac Morphology

**FIGURE 1:**
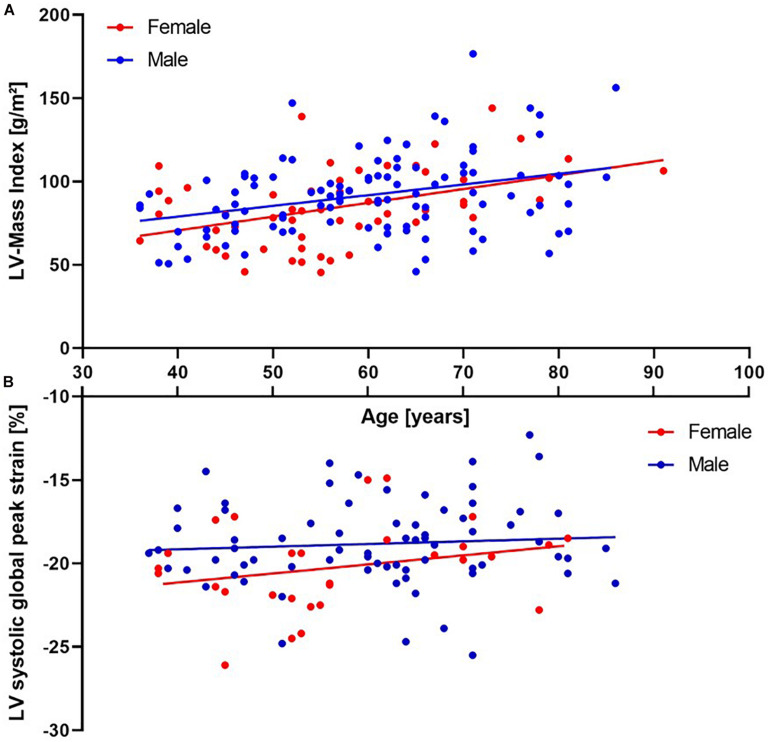
(**A**, upper panel) – scatter plot of left ventricular (LV) mass index plotted against age including regression lines for groups. Note the significant increase in both sexes with age (Correlation coefficient 0.37, *p* < 0.01). Mean values do not differ between groups. (**B**, lower panel) – scatter plot of LV systolic global peak strain plotted against age including regression lines for groups. Strain values do not correlate with age in both groups, whereas mean values differ significantly between groups (*p* = 0.01).

As listed in Table 1, male athletes had greater intraventricular septal diameter (*p* < 0.01), LV diameter (*p* < 0.01) and posterior wall thickness (*p* = 0.049) when measured in end-diastole, compared with women. This resulted in a significantly greater LV mass in men (174 ± 4 vs 141 ± 36 *g*, *p* < 0.01). However, when indexing to body surface area, LV mass index did not differ between sexes (*p* = 0.07, Figure 1). Both, LV biplanar volumes and right ventricular areas, derived from the apical four chamber view, were larger in male athletes than in female athletes, not only in systole but also in diastole (*p* < 0.05 for all; Table 1). Furthermore, this difference remained significant even after indexing to body surface area.

### Left Ventricular Systolic Function

Left ventricular systolic function assessed by biplanar ejection fraction did not differ between sexes (*p* = 0.06; Table 1). However, when cardiac strain analysis was utilized, women had a higher LV systolic global longitudinal peak strain (*p* = 0.01, Figure 1) and lower peak dispersion (*p* = 0.03) than men. Peak dispersion was defined as the standard deviation of the time until peak strain is reached within all myocardial segments and therefore, is a surrogate for the left ventricle’s economic state of systolic contraction. Stroke volume index was similar in both groups (*p* = 0.42). This was most likely due to significantly higher blood flow velocities in the LV outflow tract in women despite smaller hearts (*p* = 0.04). For the assessment of systolic right ventricular function, no differences were observed regarding TAPSE or FAC between sexes (*p* = 0.63 and *p* = 0.06, respectively).

### Left Ventricular Diastolic Function

Female athletes expressed higher intracardiac blood flow peak velocities for the early and atrial wave compared with men (*p* < 0.01 and *p* = 0.02, respectively; Table 1). However, early/atrial ratio did not significantly differ between the groups (*p* = 0.38). Furthermore, E prime (doppler velocities of the mitral annulus) was not influenced by sex (*p* = 0.19) which resulted in an increased E to E prime ratio in women (*p* = 0.01).

### Age-Related Changes

As shown in Table 2, age was associated with increases in systolic blood pressure for both male and female athletes (*r* = 0.29 and *r* = 0.37, both *p* < 0.01). Septal and posterior wall diameters were associated with age for both men (*r* = 0.27, *p* < 0.01) and women (*r* = 0.51, *p* < 0.01). This may explain the finding of a strong positive correlation between age and LV mass index for both groups (*r* = 0.33 and *r* = 0.43, both *p* < 0.01, Figure 1). Systolic function for both ventricles including ejection fraction, stroke volume index, LV systolic global longitudinal peak strain and TAPSE did not demonstrate significant correlations with age in both sexes. However, when combining both men and women, TAPSE was found to have a negative correlation with age (*p* = 0.04). Moreover, LV contraction became more desynchronized with age, as measured by peak dispersion.

**TABLE 2:** Associations of cardiovascular metrics with age in male and female masters athletes.

Variable	Males		Females		All	
			
	Correlation coefficient	*P*	Correlation coefficient	*P*	Correlation coefficient	*P*
**Hemodynamics**						
Heart rate	0.18	0.08	–0.01	0.96	0.09	0.25
Systolic blood pressure	0.29	<0.01	0.38	<0.01	0.33	<0.01
Diastolic blood pressure	0.13	0.18	0.22	0.11	0.17	0.03
Mean arterial pressure	0.24	0.01	0.29	0.04	0.27	<0.01
Cardiac output	0.21	0.06	0.02	0.89	0.17	0.06
Systemic vascular resistance	–0.10	0.38	0.03	0.84	–0.51	0.58
**Ventricular morphology**						
IVSd	0.27	<0.01	0.51	<0.01	0.36	<0.01
LVend-diastolic diameter	–0.09	0.36	–0.14	0.31	–0.07	0.36
LV posterior wall diameter	0.31	<0.01	0.47	<0.01	0.37	<0.01
LVmass	0.27	<0.01	0.39	<0.01	0.32	<0.01
LVmass index	0.33	<0.01	0.43	<0.01	0.37	<0.01
LVend-systolic volume index	–0.20	0.04	–0.12	0.39	–0.12	0.13
LV end-diastolic volume index	–0.04	0.72	–0.06	0.65	–0.02	0.79
RV end-systolic area index	0.20	0.03	0.06	0.69	0.20	0.02
RV end-diastolic area index	0.02	0.86	–0.06	0.67	0.04	0.66
**Systolic function**						
LV ejection fraction	0.16	0.12	–0.01	0.94	0.09	0.30
RV fractional area change	–0.27	<0.01	–0.01	0.50	–0.23	<0.01
TAPSE	–0.18	0.06	–0.14	0.31	–0.16	0.04
LV peak dispersion	0.36	<0.01	0.37	0.05	0.37	<0.01
LVsystolic global longitudinal peak strain	–0.06	0.61	–0.20	0.29	–0.13	0.18
LV OT mean velocity	–0.05	0.62	0.30	0.14	–0.12	0.14
LV OT stroke volume index	0.06	0.57	0.07	0.67	0.07	0.43
**LV Diastolic function**						
MV early filling wave (E)	–0.29	<0.01	–0.29	0.03	–0.31	<0.01
MVatrial filling wave (A)	0.34	<0.01	0.08	0.57	0.23	<0.01
E/A Ratio	–0.44	<0.01	–0.30	0.03	–0.39	<0.01
MVaveraged e prime	–0.58	<0.01	–0.80	<0.01	–0.66	<0.01
E/averaged e prime ratio	0.27	<0.01	0.45	<0.01	0.32	<0.01

*IVSd, intraventricular septal diameter; LV, left ventricular; RV, right ventricular; TAPSE, tricuspid annulus plane systolic excursion; OT, outflow tract, MV, mitral valve; and e prime, Doppler velocities of the mitral annulus.*

Diastolic function was inversely associated with age for both male and female athletes, as seen by a decrease in the early filling wave (*r* = −0.29, *p* < 0.01 and *r* = −0.29, *p* = 0.03) and an increase in E to E prime ratio (*r* = 0.27 and *r* = 0.45, both *p* < 0.01). Atrial filling wave was associated with age in male athletes (*r* = 0.34, *p* < 0.01), but not in female athletes.

Moreover, when analyzing the aforementioned parameters by individual age groups (35–44 years, 45–54 years, 55–64 years, 65–74 years, and >74 years) for both sexes, we observed similar trends as seen in previous correlations analysis. Supplementary Table 1 shows the results of the age stratification analysis.

### Cardiac Pathologies

We observed a number of asymptomatic cardiac abnormalities during echocardiography. This includes LV-hypertrophy, atrial dilation, and different valve deficiencies. Furthermore, we found 1 case with dilated and 3 cases with hypertrophic cardiomyopathy as well as one case with suspected cardiac storage disease. Details of observed pathologies can be found within Supplementary Table 2.

## Discussion

This is the first and largest study to investigate sex differences in cardiac function and morphology via echocardiography in male and female masters athletes on an international scale. We found that female masters athletes exhibited smaller left ventricles but compensated for reduced cardiac volume by increased LV filling pressures resulting in higher intracardiac blood flow velocities and stronger contractions displayed by global strain analysis. Additionally, deterioration of systolic and/or diastolic cardiac function in male and female athletes was found to be absent with aging. This effect is even more pronounced as we could observe morphological adaptation trending toward LV hypertrophy.

### Hemodynamics and Morphology

Similar to previous studies in sedentary adults (Lloyd-Jones et al., 2005), increasing age was associated with elevations in systolic blood pressure whereas no association was observed between age and diastolic blood pressure. The sex differences in systolic and diastolic blood pressure were not found in the present study. Although we cannot characterize the load on the left ventricle, imposed by the arterial system, both MAP and SVR were not different between sexes. In general, both male and female masters athletes (70.9%/60.0%, Supplementary Table 2) showed concentric LV hypertrophy with increased LV thickness and LV cavity. This finding is in agreement with typical morphological alterations seen in the athlete’s heart (Fagard, 2003). On average, male athletes possessed 23% greater LV mass than female athletes. After normalizing for body surface area, the significant sex difference in LV mass disappeared. This is in contrast to general population data (Petitto et al., 2018) which demonstrated persistent sex difference in LV mass after adjustment for body size. Moreover, our data show an increase of LV mass index with increasing age with a steeper slope in women, which is in line with published data (Lieb et al., 2009).

### LV Systolic Function

Left and right ventricular systolic function and stroke volume index were not different between men and women. However, mean velocity in the LV outflow tract was elevated in women, which resulted in a significantly higher LV systolic global longitudinal peak strain when compared with men. In 119 healthy subjects (60 women, mean age 48 ± 9.3 years) Shi et al. reported a significantly higher global longitudinal strain for the endo-, mid-, and epicardial layer of the left ventricle in women between the age of 22–60 years. Beyond 60 years the effect vanishes (Shi et al., 2016). This physiological difference may be necessary for women in general, but especially for female athletes, to maintain proper stroke volume in the face of smaller ventricular volumes. Moreover, we found women to have smaller peak dispersion, which resulted in a higher economic state of ventricular contraction. Cardiac strain is a powerful predictor of cardiovascular morbidity and mortality and higher values indicate reduced individual cardiovascular risk (Haugaa and Edvardsen, 2016). Considering that postmenopausal women are known to have increased risk of cardiovascular disease development comparable to men within the general population (Rodgers et al., 2019), we hypothesize that preserved LV systolic global longitudinal peak strain in female masters athletes is a surrogate of reduced individual cardiac risk compared to men. However, future researchers should further investigate this physiological relationship.

None of the quantified LV systolic function metrics were significantly associated with age in female athletes. However, LV peak dispersion, independently associated with LV mechanical dyssynchrony and mortality (Kawakami et al., 2020), was positively correlated with age in male athletes. Additionally, FAC, also known as the percent of area change of the right ventricle between diastole and systole, was lower with age in men. FAC is associated with heart failure, stroke, and sudden cardiac death (Anavekar et al., 2008). Although these FAC values were well beyond the clinical threshold of 35% (Lang et al., 2015), these sex-related differences are consistent with the notion that males tend to suffer from cardiac diseases more frequently than women (Lloyd-Jones et al., 1999).

A comprehensive analysis of 494 subjects ranging in age from 13 to 87 years found a significant positive correlation of LV ejection fraction with age but not sex in absence of cardiovascular disease. In our study masters athletes’ ejection fraction also did not differ between sexes. However, for masters athletes we could not observe a correlation of ejection fraction with age. Furthermore, mean ejection fraction in our study was lower than compared to the corresponding age group (61.2 ± 5.0 vs 68.1 ± 5.2%; Watanabe et al., 2005).

### LV Diastolic Function

Aging is associated with marked decreases in LV diastolic function (Gates et al., 2003), and diastolic heart failure with preserved ejection fraction is increasingly prevalent among older adults (Van Heerebeek and Paulus, 2016). In the present study involving masters athletes, early filling wave and E/A ratio were inversely correlated with age. Moreover, atrial filling wave was positively associated with age in both male and female athletes. Although these findings would seem to indicate a reduction in LV diastolic function over the lifespan, lifelong exercisers such as masters athletes demonstrated relatively healthy function even in late age. Female athletes compensated smaller heart size and reduced cardiac volume with higher filling pressures as indicated by E/e prime, a surrogate metric for LV end-diastolic filling pressure. Greater filling pressures are consistent with significantly higher E and A wave in women than in men. Compared to historical age matched controls, E/A ratio in masters athletes was higher 1.29 ± 0.40 vs 1.25 ± 0.36 heralding a better diastolic function despite the significant decrease with age (Watanabe et al., 2005).

### Limitations

There are a number of study limitations that should be emphasized. First, the present study comprises of a heterogenous population. Subjects enrolled, ranged from ambitious amateurs to highly trained athletes who gathered from all over the world for an international competition. Additionally, the present study lacks a true sedentary/untrained control group for comparison and instead uses normative and clinical values referenced in accredited literature. Future studies should consider the use of a control group as it would have been valuable for comparative analysis. Second, all the testing was conducted in a field-testing laboratory set up in the stadium. Given that the focus of the study was placed on sex differences, testing conditions were identical between male and female athletes. Third, because sample size became smaller with sex-related analyses, we could not account for sport specific training or discipline such as endurance, sprint, and strength. However, these sport disciplines were equally distributed in male and female athletes. Lastly, subjects did not provide information regarding years of training experience. Future researchers should take into consideration the total number of years of training experience to further explore a dose-response relationship between exercise and cardiac function and morphology.

## Conclusion

This study aimed to elucidate cardiac function and morphology differences in male and female masters athletes. Cardiac sex differences are present even among masters athletes. Lifelong exercise training does not appear to exasperate morphological difference to a point of cardiac risk or dysfunction in both male and female athletes. In general masters athletes were found to have greater cardiac protective mechanisms when compared with a general aging population for hemodynamics, morphology, LV systolic and diastolic functions. Moreover, the deterioration of systolic and diastolic function typically associated with aging were absent in masters athletes. Therefore, this study underlines not only the safety and feasibility of life-long exercise in healthy individuals but can additionally be used to help guide exercise interventions in middle-aged and elderly to maintain cardiac function and decrease the risk of adverse cardiovascular events.

## Data Availability Statement

The raw data supporting the conclusions of this article will be made available by the authors, without undue reservation.

## Ethics Statement

This study was approved by ethic commission of Ärztekammer Nordrhein (lfd Nr 2018171) and informed consent was provided by all Human Participants. The patients/participants provided their written informed consent to participate in this study.

## Author Contributions

All authors contributed to the study conception and design. Material preparation and data collection were performed by SM, JT, and FH. Statistical analysis was done by SW and FH. The first draft of the manuscript was written by SW, HT, and FH. All authors commented on previous versions of the manuscript. All authors read and approved the final manuscript.

## Conflict of Interest

The authors declare that the research was conducted in the absence of any commercial or financial relationships that could be construed as a potential conflict of interest.
