# Supplementary material for: Age- and Sex-Differences in Cardiac Characteristics Determined by Echocardiography in Masters Athletes
Source: Front Physiol. 2021 Jan 18;11:630148. doi: 10.3389/fphys.2020.630148 (PMC7848176; doi:10.3389/fphys.2020.630148)
Supplement: Supplementary file 1 [file Table_1.DOCX]

**Supplementary Files**

**Supplementary Table 1.** Age stratification for main exemplatory endpoints.

|  |  | **Male** | | | | | | **Female** | | | | | |
| --- | --- | --- | --- | --- | --- | --- | --- | --- | --- | --- | --- | --- | --- |
| Age group | [years] | 35-44 | 45-54 | 55-64 | 65-74 | >74 | *P* | 35-44 | 45-54 | 55-64 | 65-74 | >74 | *P* |
| Sample size | [n] | 12 | 24 | 31 | 21 | 15 |  | 9 | 16 | 15 | 10 | 5 |  |
| **Hemodynamics** | |  |  |  |  |  |  |  |  |  |  |  |  |
| Heart rate at rest | [1/min] | 58 ± 9 | 54 ± 12 | 58 ± 9 | 62 ± 9 | 64 ± 13 | 0.21 | 65 ± 12 | 63 ± 12 | 61 ± 13 | 65 ± 10 | 60 ± 8 | 0.83 |
| Systolic blood pressure | [mmHg] | 127 ± 9 | 124 ± 13 | 128 ± 11 | 133 ± 13 | 137 ± 24 | 0.04 | 121 ± 13 | 124 ± 11 | 126 ± 15 | 137 ± 24 | 133 ± 21 | 0.18 |
| Diastolic blood pressure | [mmHg] | 78 ± 5 | 76 ± 7 | 80 ± 7 | 80 ± 8 | 79 ± 11 | 0.28 | 76 ± 6 | 75 ± 8 | 77 ± 9 | 80 ± 12 | 79 ± 3 | 0.66 |
| **Morphology** | |  |  |  |  |  |  |  |  |  |  |  |  |
| LV mass index | [g/m²] | 73 ± 17 | 88 ± 20 | 94 ± 17 | 96 ± 31 | 101 ± 29 | 0.02 | 80 ± 18 | 74 ± 23 | 81 ± 22 | 99 ± 22 | 107 ± 14 | 0.01 |
| **Systolic Function** | |  |  |  |  |  |  |  |  |  |  |  |  |
| LV ejection fraction | [%] | 59.2 ± 5.8 | 60.1 ± 5.8 | 60.8 ± 5.0 | 62.0 ± 4.6 | 60.0 ± 6.2 | 0.55 | 62.1 ± 3.4 | 61.2 ± 4.5 | 64.6 ± 4.5 | 62.1 ± 5.8 | 60.3 ± 5.3 | 0.34 |
| TAPSE | [mm] | 29.6 ± 3.1 | 31.0 ± 4.5 | 29.6 ± 4.3 | 29.5 ± 4.4 | 26.4 ± 4.2 | 0.03 | 31.7 ± 5.6 | 27.9 ± 4.9 | 28.5 ± 4.9 | 29.2 ± 5.9 | 29.2 ± 5.4 | 0.55 |
| LV peak dispersion | [ms] | 47 ± 8 | 53 ± 8 | 60 ± 25 | 66 ± 28 | 83 ± 47 | 0.03 | 44 ± 6 | 48 ± 8 | 54 ± 20 | 58 ± 13 | 49 ± 9 | 0.42 |
| LV systolic global longitudinal peak strain | [%] | -18.8 ± 2.1 | -19.7 ± 2.3 | -18.8 ± 2.6 | -18.8 ± 2.8 | -17.9 ± 2.8 | 0.57 | -19.8 ± 1.5 | -21.9 ± 2.7 | -18.9 ± 3.3 | -19 ± 2.6 | -20.1 ± 2.4 | 0.14 |
| LV OT stroke volume index | [ml/m²] | 44.1 ± 5.1 | 53.7 ± 8.5 | 49.7 ± 13.2 | 50.4 ± 10.7 | 49.8 ± 11.2 | 0.26 | 48.0 ± 13.4 | 48.4 ± 9.9 | 47.4 ± 9.5 | 47.4 ± 9.5 | 54.7 ± 15.0 | 0.78 |
| **LV Diastolic Function** | |  |  |  |  |  |  |  |  |  |  |  |  |
| MV early filling wave (E) | [cm/s] | 60.1 ± 30.8 | 65.8 ± 17.9 | 65.9 ± 12.4 | 60.2 ± 18.5 | 46.8 ± 23.6 | 0.04 | 85.9 ± 24.8 | 76.9 ± 18.9 | 72.5 ± 17.5 | 63.3 ± 13.7 | 73.5 ± 28.3 | 0.17 |
| MV atrial filling wave (A) | [cm/s] | 41.5 ± 21.7 | 45.7 ± 14.4 | 53.2 ± 14.1 | 58.1 ± 23.7 | 53.9 ± 30.4 | 0.09 | 58.2 ± 22.9 | 58.4 ± 16.5 | 58.1 ± 11.8 | 57.4 ± 14.2 | 63.8 ± 16.4 | 0.97 |
| E/A ratio |  | 1.47 ± 0.24 | 1.48 ± 0.30 | 1.29 ± 0.30 | 1.17 ± 0.54 | 0.91 ± 0.21 | <0.01 | 1.57 ± 0.39 | 1.43 ± 0.57 | 1.30 ± 0.42 | 1.16 ± 0.32 | 1.20 ± 0.56 | 0.33 |
| E/averaged e prime ratio |  | 4.8 ± 2.49 | 6.0 ± 1.94 | 6.48 ± 1.49 | 7.16 ± 2.36 | 5.67 ± 2.96 | 0.02 | 6.49 ± 2.32 | 6.33 ± 1.59 | 7.21 ± 1.45 | 8.19 ± 2.81 | 9.77 ± 2.5 | 0.02 |

LV= left ventricular, MV= Mitral valve

**Supplementary Table 2.** Asymptomatic Cardiac Pathologies Detected during Clinical Echocardiography in Male and Female Masters Athletes

|  |  | **Male** | | **Female** | | **Total** | |
| --- | --- | --- | --- | --- | --- | --- | --- |
|  |  | [n] | [%] of males | [n] | [%] of females | [n] | [%] |
|  |  | 103 | 65.2 | 55 | 34.8 | 158 | 100 |
| AV stenosis | °I | 1 | 1.0 | 2 | 3.6 | 3 | 1.9 |
|  | °II | 1 | 1.0 | 0 | 0.0 | 1 | 0.6 |
|  | °III | 0 | 0.0 | 0 | 0.0 | 0 | 0.0 |
| AV insufficiency | °I | 13 | 12.6 | 6 | 10.9 | 19 | 12.0 |
|  | °II | 3 | 2.9 | 1 | 1.9 | 4 | 2.5 |
|  | °III | 1 | 1.0 | 0 | 0.0 | 1 | 0.6 |
| PV stenosis | any | 0 | 0.0 | 0 | 0.0 | 0 | 0.0 |
| PV insufficiency | °II | 1 | 1.0 | 0 | 0.0 | 1 | 0.6 |
| MV stenosis | °I | 0 | 0.0 | 1 | 1.9 | 1 | 0.6 |
| MV insufficiency | °I | 28 | 27.2 | 12 | 21.8 | 40 | 25.3 |
|  | °II | 9 | 8.7 | 3 | 5.5 | 12 | 7.6 |
|  | °III | 0 | 0.0 | 0 | 0.0 | 0 | 0.0 |
|  | °IV | 0 | 0.0 | 0 | 0.0 | 0 | 0.0 |
| TV stenosis | any | 0 | 0,0 | 0 | 0.0 | 0 | 0.0 |
| TV insufficiency | °I | 36 | 35.0 | 21 | 38.2 | 57 | 36.1 |
|  | °II | 12 | 11.7 | 7 | 12.7 | 19 | 12.0 |
|  | °III | 2 | 1.9 | 0 | 0.0 | 2 | 1.3 |
|  | °IV | 0 | 0.0 | 0 | 0.0 | 0 | 0.0 |
| LV-Hypertrophy | IVSd >11(m)/  >10(f) mm | 73 | 70.9 | 33 | 60.0 | 106 | 67.1 |
| LA-Dilation | >34 ml/m² | 29 | 28.2 | 13 | 23.6 | 42 | 26.6 |
| RA-Dilation | >18 cm² | 68 | 66.0 | 16 | 29.1 | 84 | 53.2 |
| Annulo-aortic ectasia | | 2 | 1.9 | 0 | 0.0 | 2 | 1.3 |
| Dilated cardiomyopathy | | 0 | 0.0 | 1 | 1.9 | 1 | 0.6 |
| Hypertrophic cardiomyopathy | | 2 | 1.9 | 1 | 1.9 | 3 | 1.9 |
| Suspected storage disease | | 1 | 1.0 | 0 | 0.0 | 1 | 0.6 |
| Aortic fibroelastoma | | 1 | 1.0 | 0 | 0.0 | 1 | 0.6 |
| Pacemaker leads | | 1 | 1.0 | 1 | 1.9 | 2 | 1.3 |
| Persistent foramen ovale | | 0 | 0.0 | 1 | 1.9 | 1 | 0.6 |

AV= aortic valve, PV= pulmonary valve, MV= mitral valve, TV= tricuspid valve, LV= left ventricular, IVSd= interventricular septal thickness at enddiastole, m= male, f= female LA= left atrium, RA= right atrium
